# Explaining the association between frailty and mortality in older adults: The mediating role of lifestyle, social, psychological, cognitive, and physical factors

**DOI:** 10.1016/j.pmedr.2021.101589

**Published:** 2021-10-07

**Authors:** Sascha de Breij, Judith J.M. Rijnhart, Noah A. Schuster, M. Liset Rietman, Mike J.L. Peters, Emiel O. Hoogendijk

**Affiliations:** a Department of Epidemiology and Data Science, Amsterdam Public Health Research Institute, Amsterdam UMC - Location VU University Medical Center, Amsterdam, the Netherlands; b Department of Life Course and Health, National Institute for Public Health and the Environment, Bilthoven, the Netherlands; c Department of Internal Medicine, Amsterdam UMC - Location VU University Medical Center, Amsterdam, the Netherlands

**Keywords:** Mediation analysis, Frailty phenotype, Survival, Epidemiology

## Abstract

Frailty is associated with a higher risk of mortality, but not much is known about underlying pathways of the frailty-mortality association. In this study, we explore a wide range of possible mediators of the relation between frailty and mortality. Data were used from the Longitudinal Aging Study Amsterdam (LASA). We included 1477 older adults aged 65 years and over who participated in the study in 2008–2009 and linked their data to register data on mortality up to 2015. We examined a range of lifestyle, social, psychological, cognitive, and physical factors as potential mediators. All analyses were stratified by sex. We used causal mediation analyses to estimate the indirect effects in single-mediator analyses. Statistically significant mediators were then included in multiple-mediator analyses to examine their combined effect. The results showed that older men (OR = 2.79, 95% CI = 1.23;6.34) and women (OR = 2.31, 95% CI = 1.24;4.30) with frailty had higher odds of being deceased 6 years later compared to those without frailty. In men, polypharmacy (indirect effect OR = 1.21, 95% CI = 1.03;1.50) was a statistically significant mediator in this association. In women, polypharmacy, self-rated health, and multimorbidity were statistically significant mediators in the single-mediator models, but only the indirect effect of polypharmacy remained in the multiple-mediator model (OR = 1.16, 95% CI = 1.03;1.38). In conclusion, of many factors that were considered, we identified polypharmacy as explanatory factor of the association between frailty and mortality in older men and women. This finding has important clinical implications, as it suggests that targeting polypharmacy in frail older adults could reduce their risk of mortality.

## Highlights

• Frailty in older adults is associated with adverse outcomes such as mortality.
• Not much is known about underlying pathways of the frailty-mortality association.
• We assessed the mediating role of a wide range of factors.
• In both men and women, only polypharmacy was identified as explanatory factor.
• Targeting polypharmacy in frail older adults could reduce their risk of mortality.

## 1 Introduction

Because of the steep increase in the proportion of older adults in the coming years, an increase in the prevalence of frailty is expected (Hoogendijk et al., 2019). Frailty is characterized by impaired strength, slow gait, weight loss, physical inactivity, and exhaustion (Fried et al., 2001). Many studies have shown that frailty is associated with adverse outcomes such as hospitalization and mortality (Hoogendijk et al., 2019). However, it is unclear how frailty affects mortality exactly. Previous studies have shown that frailty is associated with several lifestyle, social, psychological, cognitive, and physical outcomes, e.g. sense of mastery (Dury et al., 2018), loneliness (Hoogendijk et al., 2016), polypharmacy (Herr et al., 2015), vitamin D levels (Zhou et al., 2016), cognitive impairment (Robertson et al., 2013), and depression (Soysal et al., 2017). In turn, these factors have been found to be associated with higher mortality rates (Herr et al., 2015, Gaksch et al., 2017, Infurna et al., 2013, Steptoe et al., 2013, Schultz-Larsen et al., 2008, Mykletun et al., 2009). Therefore, these factors could be acting as mediators and may explain the association between frailty and mortality. To our knowledge, no studies have been conducted in which these possible mediators have been investigated.

So far, results of studies on the effects of interventions are inconsistent and a recent meta-analysis showed there is not sufficient evidence that existing interventions can reduce the risk of adverse outcomes in community dwelling frail older adults (Van der Elst et al., 2018). A better understanding of underlying pathways, by identifying mediators, may help to develop interventions aimed at minimizing the adverse outcomes of frailty. Therefore, in this study we explore a wide range of possible mediators in the relation between frailty and mortality.

## 2 Methods

### 2.1 Sample

We used data from the Longitudinal Aging Study Amsterdam (LASA). LASA is an ongoing, prospective cohort study in the Netherlands on the determinants, trajectories and consequences of physical, cognitive, emotional, and social functioning in Dutch older adults aged 55 years or older. Measurements are conducted approximately every three years and include a main face-to-face computer assisted interview, a face-to-face computer assisted medical interview in which clinical measurements are performed and additional questions are asked, and a self-administered questionnaire. To respondents who refused to participate in a full or abbreviated face-to-face interview, a telephone interview is offered. The telephone interview takes approximately 15 min and includes a selection of key indicators of functioning. Sampling, response and procedures are described in detail elsewhere (Hoogendijk et al., 2020). LASA was approved by the medical ethics committee of the VU University Medical Center. All participants signed informed consent.

For the current study, we used data from 2008/2009 (baseline) and register data on mortality up to 2015. To measure weight loss at baseline, we also used data on weight from 2005/2006. All respondents aged 65 years and over at baseline were included in our sample (n = 1477).

### 2.2 Outcome measure

Mortality status at six year follow-up was used as the outcome measure. Data on mortality were derived from the registers of the municipalities in which the respondents were living. Since this was an explorative study, we chose to include mortality as a binary outcome (deceased no/yes) rather than a time-to-event outcome.

### 2.3 Independent variable

Frailty status at baseline was the independent variable in this study. Frailty was assessed using the five criteria of the Fried frailty phenotype: weight loss, weakness, exhaustion, low physical activity, and slow gait (Fried et al., 2001). To measure weight loss, we used data from two consecutive weight measurements with a 3-year interval. Weight loss was defined as a decrease in weight greater than 5% in the previous 3 years. Weakness was measured by the maximal hand grip strength, which was assessed with a handheld dynamometer (Takei TKK 5001, Takei Scientific Instruments, Tokyo, Japan) in a standing posture with the elbow extended. The maximal value of two measurements from the dominant hand was used. We used original cut-off points stratified by sex and body mass index to indicate weakness (Fried et al., 2001). Exhaustion was measured with two items from the Center for Epidemiologic Studies Depression Scale (CES-D (Radloff, 1977): “I felt that everything I did was an effort in the last week” and “I could not get going in the last week”. Respondents were asked how often in the last week they felt this way and response categories were (0) rarely or none of the time (<1 day), (1) some or a little of the time (1–2 days), (2) a moderate amount of the time (3–4 days), or (3) most of the time. Respondents were categorized as exhausted if they answered “2” or “3” to either of these questions. Physical activity was measured using the LASA Physical Activity Questionnaire (LAPAQ) (Stel et al., 2004). Low physical activity was defined by the lowest quintile of average time spent on walking and cycling per day during two weeks before the interview. To measure walking speed, respondents were asked to walk three meters, turn around and walk back as quickly as possible without running. Slow gait was defined by the lowest quintile, stratified by sex and height. Respondents with a frailty sum score of three and over were categorized as being frail.

### 2.4 Mediators

Continuous mediators were dichotomized if they were not normally distributed or if their association with mortality was not linear.

#### 2.4.1 Lifestyle factors

Respondents were asked how many days per week they drink alcohol and how many consumptions they drink each time. We calculated the number of glasses per day and used sex specific cut-offs to categorize alcohol use into no use (0 drinks) and moderate (men:1–3 drinks, women:1–2 drinks) to excessive use (men:4 + drinks, women:3 + drinks).

In the self-administered questionnaire, respondents were asked about their sleep problems. They were asked whether they had problems with falling asleep, waking up in the night or waking up too early in the morning, with four response categories ranging from (1) almost never to (4) almost always. Sum scores were dichotomized into no/some problems (3–5) and many problems (6–12), based on the median.

#### 2.4.2 Social factors

Loneliness was measured using the De Jong-Gierveld Loneliness Scale, which ranges from 0 to 11 (De Jong, 1999). Respondents were considered to be lonely when they had a score of 3 or higher. The size of the social network was identified by asking respondents to name the persons they were in frequent contact with and who were also important to them (0–80). Respondents were also asked how much instrumental and emotional support they received from the nine most frequently contacted persons from their social network. Response possibilities were: (1) never, (2) seldom, (3) sometimes, and (4) often. A sum score (0–36) was calculated, with higher scores reflecting more support. Sum scores were dichotomized using the median into low instrumental support (0–15) and high instrumental support (16–36) and low emotional support (0–23) and high emotional support (24–36).

Two types of social participation were included: participating in leisure activities and membership of community organizations. Respondents indicated how often ((1) almost never, (2) a few times a year, (3) every month, (4) a few times a month, (5) every week, (6) a few times a week, and (7) every day) they engaged in seven leisure activities (e.g. visiting a museum, going to a bar/restaurant, shopping for pleasure). Respondents were considered to engage in leisure activities if they engaged in at least one leisure activity at least every month (except for shopping, which had to be at least once a week). Respondents also indicated whether they were members of 12 types of community organizations, ranging from a church and sports organizations to choirs.

#### 2.4.3 Psychological factors

We measured mastery with the Pearlin Mastery Scale (Pearlin and Schooler, 1978), which consists of seven items measuring the extent to which one regards one's life-chances as being under one's own control. Answer categories range from (1) strongly disagree to (5) strongly agree. Sum scores were dichotomized into low sense of mastery (7–17) and high sense of mastery (18–35), based on the median. We assessed self-efficacy with the General Self-Efficacy Scale (GSES) (Sherer et al., 1982). In LASA, an abbreviated version was used, consisting of 12 items. These items cover three different aspects: willingness to initiate behaviour, persistence when facing adversity, and effort to complete behaviour. Answer categories ranged from (1) strongly disagree to (5) strongly agree. Sum scores were dichotomized into low self-efficacy (12–42) and high self-efficacy (43–60), based on the median.

The CES-D was used to measure depressive symptoms (Radloff, 1977). The CES-D is a 20-item self-report scale ranging from 0 to 60. We used a cut-off point of 16 to identify respondents with clinically relevant symptoms of depression (Berkman et al., 1986). Anxiety was measured with the Hospital Anxiety and Depression Scale—Anxiety scale (HADS-A) (Zigmond and Snaith, 1983), which consists of seven items. The scale ranges from 0 to 21 and a cut-off score of 8 was used to determine anxiety (Snaith, 2003).

#### 2.4.4 Cognitive factors

To measure cognitive functioning we used the Mini-Mental State Examination (MMSE) (Folstein et al., 1975). The MMSE consists of 23 items and scores range from 0 to 30. Scores were dichotomized into low cognitive functioning (0–27) and high cognitive functioning (28–30), based on the median.

In addition, respondents were asked whether they had memory complaints (no/yes).

#### 2.4.5 Physical factors

Respondents were asked if they had hypertension (no/yes). Height and weight were measured by the interviewer. Body Mass Index (BMI) was calculated and dichotomized into normal weight (<25), and overweight/obese (≥25). Only five respondents were underweight (BMI < 18.5), so this category was collapsed with the normal weight category.

To measure hearing problems, respondents were asked whether they could follow a conversation in a group of three or four persons with and without a hearing aid, and whether they could follow a conversation with one person with and without a hearing aid. Response categories were (1) yes, without difficulty, (2) yes, but with some difficulty, (3) yes, but with much difficulty, and (4) no, I cannot. Respondents were categorized as having hearing problems if they had at least some difficulty with more than one of these items. To measure vision problems, respondents were asked whether they could read small letters of the newspaper with and without glasses or contact lenses, and whether they could recognize someonés face at four meters with and without glasses or contact lenses. Response categories and categorization were the same as for hearing problems.

Multimorbidity was measured by self-reports of the following seven chronic diseases: chronic nonspecific lung disease, cardiovascular diseases, peripheral artery diseases, diabetes mellitus, stroke, osteoarthritis, and malignancies. If respondents had two or more chronic diseases they were categorized as having multimorbidity. Respondents were asked about their medication use. All medications were recoded into Anatomical Therapeutic Chemical (ATC) codes and counted. Polypharmacy was dichotomized as no (<5 medications) or yes (≥5 medications).

Self-rated health (SRH) was measured with the question ‘How is your health in general?’, with response categories ranging from (1) very good to (5) poor. Responses were dichotomized into (0) very good/good SRH or (1) less than good SRH. Pain was measured with five items from the self-administered questionnaire: “I am in pain when I am standing”, “I find it painful to change position”, “I am in pain when I am sitting”, “I am in pain when I walk”, and “I am in constant pain”. Response categories were (1) no and (2) yes. Sum scores ranged from 5 to 10 and were dichotomized into low levels of pain (5) and high levels of pain (6–10), based on the median.

Fasting blood samples were drawn and Serum 25-hydroxyvitamin D levels were determined to measure vitamin D levels. Serum 25-hydroxyvitamin D levels were standardized using the Vitamin D Standardization Program (VDSP) protocol as part of the European ODIN study (“Food-based solutions for optimal vitamin D nutrition and health through the life cycle”) (Sempos et al., 2012, Cashman et al., 2016).

### 2.5 Control variables

We included sex, age, educational level (measured by the International Standard Classification of Education 2011 (ISCED 2011)), and partner status (partner no/yes) as control variables.

### 2.6 Statistical analysis

**Fig. 1:**
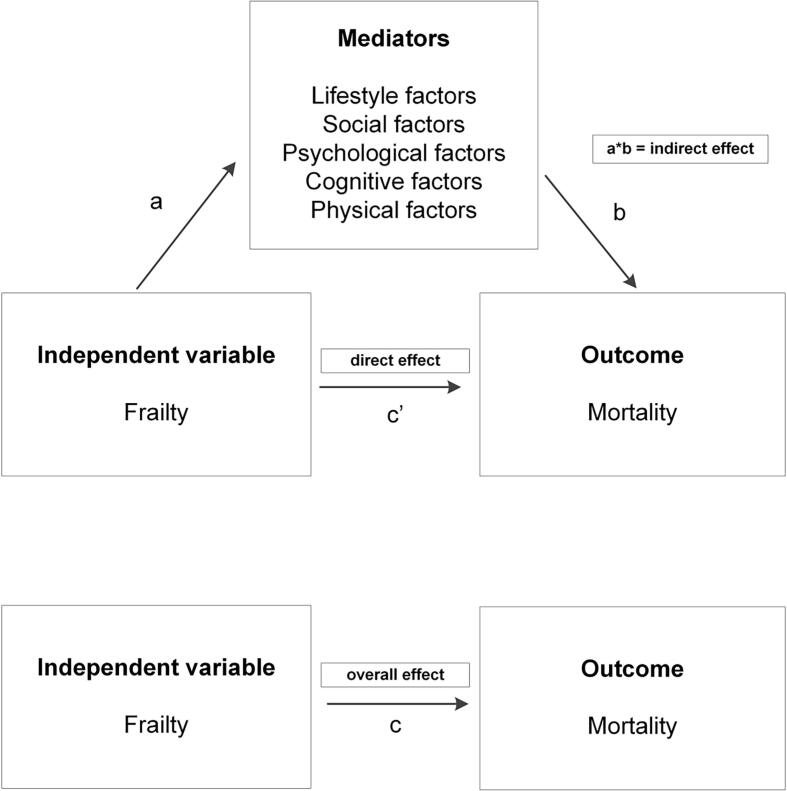
Visual representation of the mediation analyses.

Multiple imputation (MICE) was used to deal with missing values, which were assumed to be missing at random. All independent, control and outcome variables were included in the imputation process and the number of imputations was set to 30, based on the percentage of missing values (White et al., 2011). All analyses were stratified by sex and adjusted for age, educational level and partner status. First, we conducted mediation analyses with single-mediator models. We used Structural Equation Modeling (SEM) to estimate the paths visualized in Fig. 1. To estimate the c paths (overall effect of frailty on mortality), b paths (effect of the mediators on mortality, while controlling for frailty), c’ paths (effect of frailty on mortality, while controlling for mediators), and a paths (effect of frailty on the mediators) in case of binary mediators, we conducted logistic regression analyses. We used causal mediation analyses to estimate the natural indirect effects (Rijnhart et al., 2019, Vanderweele, 2015). We used bootstrapping techniques (500 repetitions) to calculate the 95% confidence intervals around the indirect effects. Second, we built parallel multiple-mediator models including all mediators that were statistically significant (p < 0.05) in the single-mediator analyses. All analyses were carried out in Stata version 14.

## 3 Results

The characteristics of the sample can be found in Table 1. In men, 9.2% was considered to be frail compared to 14.6% in women. In Table 2, the estimated c paths can be found. Those who were frail had 2.79 or 2.31, for men and women respectively, times the odds of being deceased within 6 years compared to those who were not frail.

**Table 1:** Characteristics of the sample.

	Men (n = 630)			Women (n = 847)		
	Not frail	Frail	Total	Not frail	Frail	Total
Frail (%)			9.2			14.6
Deceased within 6 years (%)	20.7	69.0	25.2	19.0	62.9	25.4
Age (M(SD))	74.5 (6.9)	84.5 (7.7)	75.5 (7.6)	75.9 (7.5)	84.4 (8.1)	77.2 (8.2)
Educational level (M(SD)) Range 1–9	4.6 (2.1)	3.8 (1.9)	4.5 (2.1)	3.6 (1.9)	3.0 (1.6)	3.5 (1.9)
Partner status: with partner (%)	84.0	61.7	81.9	49.4	23.2	45.6
Sleep problems: above median (%)	43.7	62.1	45.4	66.3	76.1	67.8
Alcohol use: moderate/excessive use	80.4	65.5	79.0	61.0	40.1	57.9
Loneliness (%)	30.6	48.4	32.2	32.0	52.3	35.0
Network size (M(SD)) Range 0–80	15.1 (9.7)	10.4 (7.2)	14.7 (9.6)	16.2 (9.6)	12.0 (8.2)	15.6 (9.5)
Instrumental support received: above median (%)	47.4	47.1	47.4	48.5	58.4	49.9
Emotional support received: above median (%)	37.8	31.9	37.3	56.6	51.1	55.8
Participating in leisure activities (%)	77.8	40.3	74.4	67.8	36.2	63.2
Member of community organizations (%)	88.2	67.2	86.2	82.3	62.7	79.5
Mastery: above median (%)	57.6	33.4	55.4	50.6	20.4	46.2
Self-efficacy: above median (%)	58.3	28.8	55.6	41.1	23.5	38.5
Depression (%)	8.6	37.2	11.2	13.9	45.7	18.6
Anxiety (%)	5.8	16.0	6.8	8.8	19.6	10.4
Memory complaints (%)	35.0	55.7	36.9	33.4	46.2	35.3
MMSE score: above median (%)	57.8	25.0	54.8	57.0	24.8	52.3
Hypertension (%)	38.0	21.2	36.5	32.4	26.7	31.5
BMI: overweight/obese (%)	71.0	64.7	70.5	68.8	75.8	69.9
Polypharmacy (%)	31.5	69.5	35.0	33.6	65.0	38.2
Hearing problems (%)	18.2	43.3	20.5	17.8	46.2	22.0
Vision problems (%)	67.9	74.1	68.5	78.4	85.6	79.5
Multimorbidity (%)	40.8	71.7	43.7	42.2	71.3	46.5
Poor self-rated health (%)	28.7	72.4	32.7	39.2	64.5	42.9
Pain: above median (%)	24.2	50.9	26.6	36.5	63.5	40.4
Vitamin D: standardized 25-hydroxyvitamin D levels (M(SD))	62.6 (21.6)	51.4 (19.7)	61.6 (21.7)	60.7 (22.5)	50.5 (19.1)	59.2 (22.3)

**Table 2:** Total effect of frailty on 6-year mortality (c path).

	Men OR (95%CI)	Women OR (95%CI)
Crude c path	7.48 (3.65;15.33)**	5.69 (3.35;9.67)**
Adjusted c path^a^	2.79 (1.23;6.34) *	2.31 (1.24;4.30) **

a adjusted for age, educational level, and partner status.

* p < 0.05

** p < 0.01

The results of the single-mediator analyses are reported in Table 3. In men, polypharmacy was found to be a mediator in the association between frailty and mortality. Frail men were at higher risk of polypharmacy (OR = 3.68, 95%CI = 1.64;8.25) than men who were not frail. In turn, polypharmacy increased the risk of mortality (OR = 1.82, 95%CI = 1.10;8.25). The indirect effect of 1.17 indicates that men who are frail have 1.17 times the odds of mortality due to higher odds of polypharmacy compared to men who are not frail. In women, we found mediating effects for polypharmacy (OR = 1.15, 95%CI = 1.04;1.38), multimorbidity (OR = 1.18, 95%CI = 1.04;1.45), and self-rated health (OR = 1.06, 95%CI = 1.00;1.21).

**Table 3:** Analysis of mediators of the association between frailty and 6-year mortality: single-mediator models.

		Men				Women		
Mediator	a path OR (95%CI)	b path OR (95%CI)	c’ path OR (95%CI)	Indirect effect OR (95%CI)	a path OR (95%CI)	b path OR (95%CI)	c’ path OR (95%CI)	Indirect effect OR (95%CI)
*Lifestyle*								
Sleep problems	2.75 (1.26;6.01) *	0.70 (0.42;1.17)	3.25 (1.36;7.73) **	0.94 (0.81;1.02)	1.09 (0.57;2.07)	1.00 (0.58;1.74)	2.17 (1.11;4.24) *	1.00 (0.97;1.03)
Alcohol use (no vs moderate/excessive use)	0.72 (0.31;1.65)	0.58 (0.33;1.01)	2.63 (1.15;6.03) *	1.02 (0.93;1.10)	0.51 (0.29;0.88) *	0.73 (0.45;1.18)	2.24 (1.20;4.18) *	1.02 (0.99;1.08)
*Social*								
Loneliness	1.06 (0.48;2.35)	1.78 (1.06;2.97) *	2.81 (1.23;6.42) *	1.00 (0.94;1.10)	1.47 (0.85; 2.55)	1.34 (0.82;2.19)	2.26 (1.21;4.21) *	1.02 (0.98;1.09)
Network size (a path: B (95%CI))	−1.36 (-4.87;2.16)	0.94 (0.90;0.97) **	2.74 (1.19;6.34) *	1.09 (0.91;1.32)	−0.46 (-2.90;1.97)	0.96 (0.93;0.99) *	2.34 (1.26;4.36) **	1.02 (0.94;1.12)
Instrumental support received	1.04 (0.49;2.21)	0.83 (0.51;1.37)	2.71 (1.18;6.24) *	1.00 (0.96;1.03)	1.70 (0.96;2.99)	0.75 (0.45;1.25)	2.40 (1.24;4.62) **	0.98 (0.90;1.02)
Emotional support received	0.93 (0.42;2.08)	0.69 (0.41;1.18)	2.62 (1.13;6.09) *	1.00 (0.96;1.05)	1.57 (0.88;2.79)	0.75 (0.45;1.25)	2.39 (1.24;4.61) **	0.98 (0.92;1.02)
Participation in leisure activities	0.30 (0.14;0.67) **	0.90 (0.47;1.70)	2.71 (1.17;6.28) *	1.01 (0.96;1.09)	0.68 (0.37;1.24)	0.41 (0.25;0.69) **	2.18 (1.15;4.13) *	1.04 (0.97;1.12)
Membership of community organizations	0.36 (0.07;1.88)	0.38 (0.10;1.49)	2.64 (1.14;6.11) *	1.14 (0.65;1.50)	1.54 (0.38;6.19)	0.22 (0.06;0.76) *	2.53 (1.33;4.80) **	0.87 (0.39;1.09)
*Psychological*								
Mastery	0.70 (0.32;1.55)	1.01 (0.61;1.67)	2.75 (1.19;6.34) *	1.00 (0.96;1.03)	0.32 (0.17;0.62) **	0.92 (0.55;1.55)	2.31 (1.20;4.45) *	1.01 (0.96;1.06)
Self-efficacy	0.39 (0.18;0.89) *	1.20 (0.72;2.00)	2.82 (1.22;6.55) *	0.98 (0.93;1.03)	0.78 (0.43;1.44)	0.93;0.55;1.59)	2.35 (1.23;4.48) *	1.00 (0.97;1.03)
Depression	5.01 (1.97;12.77) **	1.41 (0.63;3.15)	2.61 (1.13;6.00) *	1.12 (0.84;1.48)	4.31 (2.33;8.00) **	1.14 (0.61;2.14)	2.23 (1.18;4.21) *	1.04 (0.85;1.31)
Anxiety	4.67 (1.52;14.33) **	1.27 (0.49;3.29)	2.71 (1.19;6.20) *	1.08 (0.78;1.51)	3.65 (1.69;7.89) **	1.54 (0.71;3.35)	2.22 (1.20;4.14) *	1.12 (0.89;1.60)
*Cognitive*								
Memory complaints	2.14 (1.02;4.51) *	1.14 (0.69;1.86)	2.71 (1.18;6.21) *	1.02 (0.95;1.09)	1.55 (0.90;2.78)	1.07 (0.65;1.77)	2.29 (1.23;4.28) **	1.00 (0.97;1.06)
MMSE score	0.71 (0.31;1.62)	0.78 (0.47;1.31)	2.75 (1.21;6.26) *	1.01 (0.97;1.06)	0.46 (0.26;0.81) **	0.66 (0.40;1.08)	2.18 (1.17;4.08) *	1.03 (0.99;1.09)
*Physical*								
Hypertension	2.51 (1.18;5.33) *	1.09 (0.66;1.82)	2.73 (1.19;6.25) *	1.02 (0.92;1.14)	0.80 (0.47;1.38)	0.83 (0.52;1.34)	2.31 (1.24;4.30) **	1.00 (0.98;1.03)
BMI: overweight/obese	0.71 (0.32;1.54)	0.74 (0.43;1.26)	2.81 (1.23;6.42) *	1.01 (0.97;1.07)	1.59 (0.83;3.03)	0.64 (0.38;1.07)	2.33 (1.24;4.39) **	0.97 (0.86;1.01)
Polypharmacy	3.68 (1.64;8.25) **	1.82 (1.10;3.00) *	2.34 (1.02;5.35)	1.17 (1.02;1.48) *	2.50 (1.42;4.39) **	2.40 (1.47;3.90) **	1.88 (0.99;3.56)	1.15 (1.04;1.38) *
Hearing problems	1.83 (0.80;4.16)	0.90 (0.48;1.69)	2.82 (1.24;6.43) *	0.99 (0.87;1.08)	1.76 (0.95;3.26)	1.40 (0.79;2.49)	2.23 (1.19;4.17) *	1.04 (0.98;1.13)
Vision problems	1.69 (0.73;3.94)	1.12 (0.66;1.91)	2.75 (1.21;6.26) *	1.01 (0.93;1.09)	1.79 (0.83;3.86)	1.06 (0.58;1.95)	2.30 (1.24;4.29) **	1.01 (0.94;1.11)
Multimorbidity	3.67 (1.50;8.94) **	1.54 (0.94;2.53)	2.47 (1.08;5.65) *	1.12 (1.00;1.37)	3.48 (1.87;6.47) **	2.05 (1.24;3.39) **	1.88 (0.99;3.55)	1.18 (1.04;1.45) *
Poor self-rated health	7.75 (3.27;18.33) **	1.40 (0.83;2.37)	2.42 (1.03;5.68) *	1.16 (0.91;1.54)	2.07 (1.19;3.57) *	1.65 (1.02;2.66) *	2.12 (1.13;3.95) *	1.06 (1.00;1.21) *
Pain	3.37 (1.52;7.44) **	1.51 (0.88;2.61)	2.85 (1.19;6.88) *	1.11 (0.99;1.37)	4.48 (2.31;8.68) **	1.08 (0.64;1.82)	2.21 (1.10;4.47) *	1.02 (0.88;1.24)
Vitamin D (a path: B (95%CI))	−4.44 (-17.07;5.18)	0.99 (0.97;1.00)	2.68 (1.16;6.18) *	1.06 (0.99;1.15)	−3.13 (-10.15;3.89)	0.98 (0.96;0.99) *	2.19 (1.16;4.12) *	1.07 (1.00;1.17)

^a^ adjusted for age, educational level, and partner status.

* p < 0.05

** p < 0.01

Next, we built a multiple-mediator model (Supplementary material, Supplementary Table 1), to explore whether these mediating effects in women were independent of each other and to examine the total indirect effect. Only the mediating effect of polypharmacy remained statistically significant (mediator-specific indirect effect OR = 1.16, 95%CI = 1.03;1.38), indicating that polypharmacy is a mediator even after controlling for multimorbidity and self-rated health. The indirect effect of multimorbidity (mediator-specific indirect effect OR = 1.11, 95%CI = 0.95;1.30), however, could be explained by polypharmacy and self-rated health and the indirect effect of self-rated health (mediator-specific indirect effect OR = 1.04, 95%CI = 0.95;1.18) could be explained by polypharmacy and multimorbidity.

## 4 Discussion

This study among community-dwelling older adults in the Netherlands was, to our knowledge, the first to explore a wide range of mediators of the association between frailty and mortality. We observed, just like in previous studies (Hoogendijk et al., 2019), that in both men (OR = 2.79, 95%CI = 1.23;6.34) and women (OR = 2.31, 95%CI = 1.24;4.30), frailty was associated with a risk of mortality six years later. However, we also extended the previous literature by identifying factors that may explain this association. Especially, polypharmacy seems to play an important role in the frailty-mortality relationship.

We found that in men polypharmacy mediated the association between frailty and mortality. In women, polypharmacy, multimorbidity, and self-rated health were mediators in the single-mediator analyses. However, in the multiple-mediator model, only the mediating effect of polypharmacy remained, indicating that the indirect effects through multimorbidity and self-rated health are explained by polypharmacy. Approximately one third of the older adults in our total sample had polypharmacy and those who were frail had much higher odds of having polypharmacy compared to those who were not frail. Polypharmacy is indeed common among older adults with frailty, with a recent meta-analysis by Palmer and colleagues reporting a prevalence of 59% (Palmer et al., 2019). Polypharmacy in frail older adults has been shown to be associated with adverse outcomes, e.g. hospitalization and mortality (Bonaga et al., 2018) and is most often chronic (Wastesson et al., 2019). Research on deprescribing shows that reducing the number of prescribed medications may reduce adverse outcomes (Liacos et al., 2020). Patient engagement and shared decision making are thought to be important aspects of successful deprescribing (Liacos et al., 2020, Reeve et al., 2014, Stewart et al., 2017). Thus, deprescribing in older adults with frailty is important to reduce adverse outcomes. The widespread adoption of standard guidelines such as from the Screening Tool of Older Person’s Prescriptions (STOPP (Gallagher et al., 2008) of Beer’s Criteria (Society AG and Updated, 2019) is recommended (Dent et al., 2019).

A remarkable finding is that of the broad range of factors considered in the current analysis, most factors were not identified as a mediator of the frailty-mortality association. This applies to all factors in the social, psychological, lifestyle, and cognitive domain. This contradicts hypotheses previously formulated in the literature. For example, is has been suggested that social factors and psychosocial resources play an important role in explaining the association between frailty and adverse health outcomes (Mehrabi and Béland, 2020, Dent and Hoogendijk, 2015). However, none of these factors were identified as mediators in our analyses. One explanation for the limited number of identified mediators could be that we missed important factors in our study, such as inflammatory markers and other biomarkers. Another explanation is that we are investigating a group that is already too far in the trajectory leading to adverse outcomes. Frailty is the outcome of a complex process of biological aging (Hoogendijk et al., 2019), which may be reversed (Gwyther et al., 2018, Ambagtsheer et al., 2019). However, the factors considered in the current study may play a more important role in earlier life stages as determinant of frailty and therefore do not turn up as mediators in the current analysis.

Uncovering mediating effects provides us insight in the pathways leading from frailty to mortality, which could ultimately contribute to public health strategies aimed at reducing the adverse outcomes of frailty. This is much needed, as there is not sufficient evidence that existing interventions to prevent adverse outcomes in frail older people are effective (Van der Elst et al., 2018). Although we were not able to identify multiple mediators of the frailty-mortality association, we identified polypharmacy as an important mediator. Polypharmacy can be reduced by adopting the practical guidelines mentioned above. Therefore, intervening on polypharmacy might be a successful strategy in reducing the mortality risk of frail older adults.

Our study has some limitations. First, frailty and the mediators were all measured at the same point in time. Because of this, we cannot make any statements about causation. For example, it is possible that frailty is an outcome of polypharmacy rather than the other way around (Rolland and Morley, 2016), although most likely, this relationship is bidirectional (Gutiérrez-Valencia et al., 2018). It is well-known that multimorbidity and associated polypharmacy, functional impairment and frailty co-exist (e.g., (Bonaga et al., 2018), and that the order in which these entities develop could vary between older adults. Not everybody with multimorbidity develops frailty, and not everybody with frailty has multimorbidity (Vetrano et al., 2019). One of the most important features of the frailty concept is that it encompasses a state of increased vulnerability to adverse outcomes in case of stressor events. The main reason for including polypharmacy as a potential mediator in our analysis is that polypharmacy is a potential stressor in people who are already frail. Older adults with frailty lack the physiological reserves to cope with the side effects of polypharmacy, such as drug interactions, adverse drug events and functional decline, which in turn increases the risk of mortality.

Second, there is a slight overlap between our measure of frailty and one of the mediators: depression. The exhaustion items from the CES-D are used in both measures. It could be that the association between frailty and depression is an overestimation because of this overlap. However, the depression scale also consists of 18 other items and results are unlikely to be affected by this overlap, since other measures of frailty produce similar results (Soysal et al., 2017). Third, in our mediation models, we only included age, educational level, and partner status as confounders. However, the factors we explored as possible mediators may also act as confounders or suppressors. Non-significant indirect effects may be due to variables suppressing these effects (MacKinnon et al., 2000). Thus, it is recommended that future research, with possibly larger sample sizes, also examines the role of the factors under investigation as confounders/suppressors. Fourth, other factors which we did not have available in the LASA dataset should be explored as possible mediators, e.g. inflammation, falls, food intake. Fifth, it would be interesting to investigate whether the mediators found in the current study are also mediators of the association between frailty and other adverse outcomes, e.g. hospitalization or morbidity, or whether other mediators play a role in these associations. Sixth, it is important to note that our study was an explorative one. Future research, with preplanned hypotheses, should be conducted to confirm our observed associations. Finally, while we focused on mortality as a relative short-term outcome, future research investigating long-term associations between frailty and mortality, using survival analyses, could give additional insight into the mechanisms at play.

In conclusion, of the broad range of factors considered in the current study, polypharmacy was the only identified explanatory factor of the association between frailty and mortality in older adults. By targeting polypharmacy, the negative effect of frailty on mortality may be reduced. Further research is needed to confirm and expand our findings.

**Data availability**

The data underlying the results presented in this study are available from the Longitudinal Aging Study Amsterdam (LASA). Data of LASA may be requested for research purposes. More information on data requests can be found on the LASA website: www.lasa-vu.nl.

## Funding

EH was supported by an NWO/ZonMw Veni fellowship [Grant Number 91618067]. The Longitudinal Aging Study Amsterdam (LASA) is largely supported by a grant from the Netherlands Ministry of Health Welfare and Sports, Directorate of Long-Term Care.

## CRediT authorship contribution statement

**Sascha de Breij:** Conceptualization, Methodology, Formal analysis, Writing – original draft, Writing – review & editing. **Judith J.M. Rijnhart:** Conceptualization, Methodology, Writing – review & editing. **Noah A. Schuster:** Conceptualization, Writing – review & editing. **M. Liset Rietman:** Writing – review & editing. **Mike J.L. Peters:** Writing – review & editing. **Emiel O. Hoogendijk:** Conceptualization, Writing – review & editing, Supervision.

## Declaration of Competing Interest

The authors declare that they have no known competing financial interests or personal relationships that could have appeared to influence the work reported in this paper.
