# Supplementary material for: Explaining the association between frailty and mortality in older adults: The mediating role of lifestyle, social, psychological, cognitive, and physical factors
Source: Prev Med Rep. 2021 Oct 7;24:101589. doi: 10.1016/j.pmedr.2021.101589 (PMC8683887; doi:10.1016/j.pmedr.2021.101589)
Supplement: Supplementary Data 1 [file mmc1.docx]

|  |  |  |  |  |
| --- | --- | --- | --- | --- |
| Mediator | **a path**  **OR (95%CI)^a^** | **b path**  **OR (95%CI)^a^** | **c’ path**  **OR (95%CI)^a^** | **Indirect effect**  **OR (95%CI)^a^** |
| Polypharmacy | 2.50 (1.42;4.39)** | 1.99 (1.18;3.37)* | 1.69 (0.89;3.22) | 1.16 (1.03;1.38)* |
| Multimorbidity | 3.48 (1.87;6.47)** | 1.47 (0.84;2.60) | 1.69 (0.89;3.22) | 1.11 (0.95;1.30) |
| Poor self-rated health | 2.07 (1.19;3.57)* | 1.25 (0.75;2.11) | 1.69 (0.89;3.22) | 1.04 (0.95;1.18) |

**SUPPLEMENTARY MATERIAL**

**Supplementary Table 1.** Multiple mediator model (women)

^a^ adjusted for age, educational level, and partner status

* p<0.05; ** p<0.01

**Stata syntax used for the multiple mediator model:**

mi convert wide, clear

mi estimate, cmdok: gsem (deceased_6y <- frailty multimorbidity polypharmacy srh age_cent education_cent partner_cent, latent(nocapslatent) family(binomial) link(logit))(multimorbidity <- frailty age_cent education_cent partner_cent, latent(nocapslatent) family(binomial) link(logit)) (polypharmacy <- frailty age_cent education_cent partner_cent, latent(nocapslatent) family(binomial) link(logit)) (srh <- frailty age_cent education_cent partner_cent, latent(nocapslatent) family(binomial) link(logit))

*Use matrix command to check position of coefficients

matrix list e(b_mi)

gen a1 = el(e(b_mi),1,9) /*exposure-mediator1 effect estimate*/

gen a2 = el(e(b_mi),1,14) /*exposure-mediator2 effect estimate*/

gen a3 = el(e(b_mi),1,19) /*exposure-mediator3 effect estimate*/

gen im1 = el(e(b_mi),1,13) /*Intercept of the mediator1 model*/

gen im2 = el(e(b_mi),1,18) /*Intercept of the mediator2 model*/

gen im3 = el(e(b_mi),1,23) /*Intercept of the mediator3 model*/

gen b1 = el(e(b_mi),1,2) /*mediator1-outcome effect estimate*/

gen b2 = el(e(b_mi),1,3) /*mediator2-outcome effect estimate*/

gen b3 = el(e(b_mi),1,4) /*mediator3-outcome effect estimate*/

gen ab1 = ((1+exp(im1))*(1+exp(b1+im1+a1)))/((1+exp(im1+a1))*(1+exp(b1+im1)))

di ab1

gen ab2 = ((1+exp(im2))*(1+exp(b2+im2+a2)))/((1+exp(im2+a2))*(1+exp(b2+im2)))

di ab2

gen ab3 = ((1+exp(im3))*(1+exp(b3+im3+a3)))/((1+exp(im3+a3))*(1+exp(b3+im3)))

di ab3

** BOOTSTRAP **

capture program drop mediate_bootstrap2

program define mediate_bootstrap2, rclass

syntax [if]

mi estimate, cmdok: gsem (deceased_6y <- frailty multimorbidity polypharmacy srh age_cent education_cent partner_cent, latent(nocapslatent) family(binomial) link(logit))(multimorbidity <- frailty age_cent education_cent partner_cent, latent(nocapslatent) family(binomial) link(logit)) (polypharmacy <- frailty age_cent education_cent partner_cent, latent(nocapslatent) family(binomial) link(logit)) (srh <- frailty age_cent education_cent partner_cent, latent(nocapslatent) family(binomial) link(logit))

return scalar ab1= ((1+exp(el(e(b_mi),1,13)))*(1+exp(el(e(b_mi),1,2)+el(e(b_mi),1,13)+el(e(b_mi),1,9))))/ ((1+exp(el(e(b_mi),1,13)+el(e(b_mi),1,9)))*(1+exp(el(e(b_mi),1,2)+el(e(b_mi),1,13))))

return scalar ab2= ((1+exp(el(e(b_mi),1,18)))*(1+exp(el(e(b_mi),1,3)+el(e(b_mi),1,18)+el(e(b_mi),1,14))))/ ((1+exp(el(e(b_mi),1,18)+el(e(b_mi),1,14)))*(1+exp(el(e(b_mi),1,3)+el(e(b_mi),1,18))))

return scalar ab3= ((1+exp(el(e(b_mi),1,23)))*(1+exp(el(e(b_mi),1,4)+el(e(b_mi),1,23)+el(e(b_mi),1,19))))/ ((1+exp(el(e(b_mi),1,23)+el(e(b_mi),1,19)))*(1+exp(el(e(b_mi),1,4)+el(e(b_mi),1,23))))

return scalar tot_indirect = exp(log(((1+exp(el(e(b_mi),1,13)))*(1+exp(el(e(b_mi),1,2)+el(e(b_mi),1,13)+el(e(b_mi),1,9))))/ ((1+exp(el(e(b_mi),1,13)+el(e(b_mi),1,9)))*(1+exp(el(e(b_mi),1,2)+el(e(b_mi),1,13))))) + log(((1+exp(el(e(b_mi),1,18)))*(1+exp(el(e(b_mi),1,3)+el(e(b_mi),1,18)+el(e(b_mi),1,14))))/ ((1+exp(el(e(b_mi),1,18)+el(e(b_mi),1,14)))*(1+exp(el(e(b_mi),1,3)+el(e(b_mi),1,18))))) + log(((1+exp(el(e(b_mi),1,23)))*(1+exp(el(e(b_mi),1,4)+el(e(b_mi),1,23)+el(e(b_mi),1,19))))/ ((1+exp(el(e(b_mi),1,23)+el(e(b_mi),1,19)))*(1+exp(el(e(b_mi),1,4)+el(e(b_mi),1,23))))))end

set more off

set seed 1234

bootstrap ab1 =r(ab1) ab2 =r(ab2) ab3 =r(ab3) tot_indirect =r(tot_indirect), reps(500):mediate_bootstrap2, level(95)

noi estat bootstrap, percentile
